# Chitinolytic Microorganisms and Their Possible Application in Environmental Protection

**DOI:** 10.1007/s00284-013-0440-4

**Published:** 2013-08-29

**Authors:** Maria Swiontek Brzezinska, Urszula Jankiewicz, Aleksandra Burkowska, Maciej Walczak

**Affiliations:** 1 Department of Environmental Microbiology and Biotechnology, Faculty of Biology and Environmental Protection, Nicolaus Copernicus University, Lwowska 1, 87-100 Toruń, Poland; 2 Department of Biochemistry, Warsaw University of Life Sciences, SGGW, 02-776 Warsaw, Poland

## Abstract

This paper provides a review of the latest research findings on the applications of microbial chitinases to biological control. Microorganisms producing these enzymes can inhibit the growth of many fungal diseases that pose a serious threat to global crop production. Currently, efforts are being made to discover producers of chitinolytic enzymes. The potential exists that natural biofungicides will replace chemical fungicides or will be used to supplement currently used fungicides, which would reduce the negative impact of chemicals on the environment and support the sustainable development of agriculture and forestry.

## Introduction

Plant diseases are a major problem facing plant cultivation and are responsible for the loss of 10 % of the total global crop production [98]. Molds, one of the most aggressive plant pathogens, are conventionally destroyed with chemical fungicides. Their widespread use, which has tripled over the last 40 years, has accelerated environmental pollution and degradation. Moreover, chemical fungicides may be lethal to beneficial insects and microorganisms populating the soil and may enter the food chain [9]. Despite their high effectiveness and ease of use, chemical fungicides have many disadvantages. Biological control, the use microorganisms to control plant diseases, offers an alternative, environmentally friendly strategy for controlling phytopathogens. Recently, biological control has been focused on microorganisms producing mycolytic enzymes, especially chitinases (CHIs), which are known to hydrolyze chitin, a major component of fungal cell walls. CHIs or chitinolytic microorganisms are currently being studied as an attractive alternative to synthetic chemicals because of their perceived safety and lower environmental impact. Biological control strategies have become an important approach for facilitating sustainable agriculture [40, 54, 80, 92].

## Occurrence of Chitin in Nature

Chitin, a β-(1,4)-linked polymer of *N*-acetyl-d-glucosamine, is one of the most abundant naturally occurring polysaccharides. Chitin occurs in combination with other polymers, such as proteins. In nature, it is found in two crystalline forms. The α-form has antiparallel chitin microfibrils with strong intermolecular hydrogen bonding and is the most abundant chitin in nature, found in shrimps and crabs. β-chitin has parallel chitin chains and occurs in squid pens [125]. Chitin is widely distributed in nature, particularly as a structural polysaccharide in fungal cell walls, the exoskeletons of arthropods, the outer shells of crustaceans, and nematodes. Approximately 75 % of the total weight of shellfish, such as shrimp, crab, and krill, is considered waste. Chitin comprises 20–58 % of the dry weight of this waste [116]. Chitin has a broad range of applications in the biochemical, food, and chemical industries and shows antimicrobial, anticholesterol, and antitumor activity [32, 77]. Chitin and its related materials are also used in wastewater treatment [26], drug delivery, and wound healing and as dietary fiber [19, 74]. Waste from the processing of marine crustaceans is a significant commercial source of chitin. Substantial amounts are produced in Asia, mainly Thailand, which is a major exporter of shrimp, and India. Utilization of waste chitin is rare, but if properly conducted, would solve the environmental problem of waste disposal and enable the great economic value of chitin to be utilized [117]. It is worth noting that the production of chitin in the marine biosphere is enormous. The estimated annual amount of chitin produced by marine zooplankton is over several billions of tons [8], and the total annual production of chitin is estimated to be 10^10^ to 10^11^ tons [31, 42]. Most of this production is in chitin shells embedded in marine zooplankton and marine crustaceans such as shrimp, crabs, and lobsters. When molted, the shed exoskeletons may contain up to five times more chitin than the body of the animal. Arthropods have the largest operational significance because they produce the greatest amount of chitin. Similarly, the surface layer of the bodies of terrestrial insects and arachnids contains considerable amounts of chitin [119]. Surprisingly, the chitin content in marine sediment is quite low. This is due to bioconversion processes carried out by marine chitinolytic bacteria [37], which transform this polysaccharide into organic compounds that are subsequently used by other microorganisms as a source of carbon and nitrogen.

## Chitinolytic Microorganisms

Commonly found in the biosphere, chitinolytic microorganisms are capable of decomposing chitin under both aerobic and anaerobic conditions. They are found in many different environments. Contrary to the expectation that they abound in environments with high amounts of chitin (such as shrimp shells), only a small number have been identified in shrimp waste: chitinolytic bacteria comprise only 4 % of the total heterotrophic bacteria. Chitinolytic fungi comprise 25–60 % of the total mold fungi, but their number is lower than the number of bacteria [106]. While investigating the correlation between shrimp waste and the number of bacteria in the digestive tract of lambs, Cobos et al. [13] identified only a small percentage of chitinolytic bacteria in this environment. They identified no chitinolytic bacteria after 30 days of feeding the lambs a diet containing no or 15 % shrimp waste. Only after 72 days of feeding the lambs on a diet containing 25 % of powdered shrimp waste did they note 0.1 % of chitinolytic bacteria in the lamb’s digestive tracts.

Chitin substrates do not always stimulate the growth of chitinolytic microorganisms. The results obtained by Zdanowski and Vosjan [123] indicate that chitinolytic microorganisms are only a small percentage of the total microbial population on krill shells. Substantially higher numbers were identified in lakes and soil within the lakes’ drainage basins. Soil and the rhizosphere are heavily colonized by chitinolytic microorganisms, and actinomycetes are the most abundant. Swiontek Brzezinska et al. [108] maintain that 45–69 % of actinomycetes and 32–40 % of mold fungi were able to decompose chitin in the soil within the drainage basin of Lake Chełmżyńskie. Because chitinolytic microorganisms isolated from soil were usually more active that chitinolytic bacteria isolated from water and bottom sediments, they could be more appropriate for agricultural use. Paul and Clark [78] report that ~90 % of all actinomycetes isolated from soil is of the genus *Streptomyces*. Almost all actinomycetes are saprophytes capable of decomposing lignin, chitin, pectin, and creatine. Soil bacteria able to degrade chitin include *Flavobacterium*, *Bacillus*, *Cytophaga*, *Pseudomonas*. Chitin-degrading fungi include, for example *Aspergillus*, *Mucor*, and *Mortierella* [90].

In aquatic environments, chitin is decomposed primarily by heterotrophic bacteria. These include aerobic bacteria of the genera *Aeromonas*, *Enterobacter*, *Chromobacterium*, *Arthrobacter*, *Flavobacterium*, *Serratia*, *Bacillus*, *Erwinia*, *Vibrio* [20, 102]. According to Swiontek Brzezinska et al. [107], 15 % of bacteria decomposed chitin in eutrophic Lake Chełmżyńskie. However, in the bottom sediments of this lake, a much lower number of chitinolytic microorganisms were identified. Mudryk [71], in surface water of Lake Gardno, found that 10.6 % of bacteria degraded chitin, compared with only 5 % in sediment.

Chitinolytic microorganisms inhabit a wide range of environments. Kopečny et al. [56] found chitinolytic bacteria in the feces of wild herbivores (e.g., bison—*Bibos bona*sus, llama**—** *Llama vicugna* *paca*, and elk**—** *Elaphurus davidianus*) and domestic herbivores (e.g., sheep and cow). They were also found in the rumen fluid of cows, which are unable to produce enzymes for digesting chitin and thus offer a living environment for chitinolytic bacteria in exchange for help digesting this tough compound. The majority of the identified bacteria belonged to the genus *Clostridium*, whose most common strain, *Clostridium* sp. ChK5, decomposes colloidal chitin and produces acetate, a salt of butyric acid, and lactate.

There is little information on the participation of anaerobic microorganisms in the degradation of chitin, although chitynolityc bacteria of the genus *Clostridium* have been described in marine environments [8, 110] and Gram-negative, anaerobic bacteria are present in sediment [79]. The results obtained by Reguera and Leschine [84] suggest that the use of chitin as a source of nitrogen may be widely distributed among anaerobic and aerobic cellulolytic microbes. *Cellulomonas* (ATCC 21 399) is capable of rapid degradation of cellulose and chitin both aerobically and anaerobically. Sturz and Robinson [99] observed that the degradation of chitin occurs mainly in surface sediments, where aerobic bacteria dominate and play a decisive role in its degradation.

## Chitinolytic Enzymes

CHIs are glycoside hydrolases that catalyze the decomposition of chitin. They are produced by microorganisms, fungi, insects, plants, and animals and are also found in human blood serum [30]. They hydrolyze the β-1,4-glycosidic bonds between the *N*-acetyl-d-glucosamine residues that comprise a chitin chain [41]. Complete enzymatic hydrolysis of chitin to free *N*-acetylglucosamine is performed by a chitinolytic system consisting of a diverse group of enzymes that catalyze the hydrolytic polymerization of chitin [25, 30, 77, 91].

The nomenclature of chitinolytic enzymes is not clear. Due to the location of the hydrolyzed bond, CHIs (EC 3.2.1.14) can be divided into two categories. Endochitinases cleave chitin chains in random locations, generating low molecular weight oligomers, such as chitototetriose, chitotriose, and diacetylochitobiose. Exochitinases release chitobiose from the reducing or non-reducing end of the chitin chain. In the past, there were two other classes for these enzymes: chitobiases are responsible for the hydrolysis of chitobiose, and β-*N*-acetylglucosaminidases produce monomers of β-*N*-acetyl-d-glucosamine [14, 88]. Currently, according to the nomenclature established by the Nomenclature Committee of the International Union of Biochemistry and Molecular Biology, chitobiase and β-*N*-acetylglucosaminidases are included in the common set of β-*N*-acetylhexosaminidases (EC.3.2.1.52) [14, 88].

When classified by the similarity of amino acid sequences, CHIs can be divided into three families: 18, 19, and 20. Family 18 includes CHIs derived mainly from fungi but also from bacteria, viruses, animals, insects, and plants. Family 19 includes CHIs derived from plants (class I, II, and IV) and several derived from bacteria, e.g., *Streptomyces griseus*. Family 20 includes *N*-acetylglucosaminidase from *Vibrio harveyi* and *N*-acetylhexosaminidase from *Dictyostelium discoideum* and human [16, 21, 41, 77]. Many bacteria, including *Serratia marcescens*, *Aeromonas* sp*., Pseudomonas aeruginosa* K-187, and *S. griseus* HUT 6037, can synthesize several different CHIs [45, 101, 112, 116]. Moreover, the well-recognized mold fungus *Trichoderma harzianum* produces two *N*-acetylglucosaminidases, four endochitinases, and one chitobiosidase [38].

Microbial CHIs weigh from 20 to 120 kDa, with most 20–60 kDa [49, 51]. The optimum pH and temperature of CHIs are 5–8 and ~40 °C, respectively. Depending on the origin of the CHI, its activity can be inhibited or stabilized by the presence of various metal ions (Table 1). A strong inhibitor of CHIs is allosamidin, which was first reported as a specific, competitive inhibitor of insect CHI. Allosamidin has a structure similar to that of an intermediate substrate, an oxazoline ring that may be formed between the carbonyl oxygen of the *N*-acetyl group and the C-1 of *N*-acetylglucosamine during hydrolysis [55].

**Table 1:** Biochemical properties of several microbial CHIs

Microorganism	Enzyme type	Mol. wt. (kDa)	Temp. (°C)	pH	Inhibitor	Activator	Reference
*Serratia* sp. KCK	Exochitinase	57	40	5–10	Pb^2+^, Fe^2+^, Zn^2+^, Hg^2+^, Mg^2+^	–	[53]
*Pseudomonas* sp. TKU015	–	68, 30	50	5–7	Mn^2 +^, Fe^2+^, Cu^2+^, EDTA	Zn^2+^, SDS, Tween 40, Triton X-100	[118]
*Bacillus* sp. 13.26	–	60	60	7–8	Mn^2+^, Co^2+^, Ca^2+^	Mg^2+^, Ni^2+^	[122]
*Bacillus licheniformis*	–		50–70	5–6	–	Ca^2+^	[52]
*Bacillus thuringiensis* spp. colmeri 15A3	–	36	20–60	4–8	Ag^+^, Zn^2+^	Mg^2+^, Na^+^, Fe^3+^, Ca^2+^, Cu^2+^	[61]
*Bacillus subtilis* NPU 001	Endochitinase	31	50	6	Hg^2+^	–	[10]
*Bacillus brevis*	Endochitinase	85	60	8	Ag^+^	–	[62]
*Stenotrophomonas maltophilia*	Endochitinase, exochitinase	52	40–50	5–7	Hg^2+^, Cu^2+^	Ca^2+^, Mg^2+^	[46, 100]
*Sanguibacter antarcticus* KOPRI 21702T	Endochitinase	55	30–40	7.6	–	Cu^2+^, Ca^2+^, Ba^2+^	[76]
*Streptomyces* sp. DA11	–	34	50	8	Fe^2+^, Ba^2+^, EDTA, EGTA, SDS, urea	Mn^2+^, Cu^2+^, Mg^2+^	[37]
*Streptomyces halstedii* AJ-7	–	55	50	7	Hg^2+^, Ni^2+^, Pb^2+^	Co^2+^	[49]
*Streptomyces roseolus* DH	GH-18	40	60	6–8	Cu^2+^, Co^2+^, Mn^2+^	Ca^2+^, Ba^2+^, Mg^2+^	[47]
*Streptomyces venezuelae* P10	–	66	30–40	6–8	–	–	[73]
*Streptomyces aureofaciens* CMUAc130	–	40	30–40	6.5–7	Hg^2+^, Cd^2+^, Ni^2+^	Mg^2+^	[109]
*Penicillium* sp. LYG 0704	–	47	40	5	Fe^2+^, Hg^2+^	Mg^2+^, Mo^2+^	[59]
*Massilia timonae*	Endochitinase	56	55	5	Hg^2+^	Mn^2+^	[1]
	Both	64	60	Both	Both	Both	
*Trichoderma saturnisporum*	Chitobiosidase	24	60	4	–	Mn^2+^, Zn^2+^	[93]
*Trichoderma atroviride* PTCC5220	–	42	40	5	–	–	[39]
*Aspergillus fumigatus* CS-01	–	45	55	5	–	–	[121]
*Thermomyces lanuginosus* SY2	–	48	55	4.5	Fe^2+^, Ag^+^, Hg^2+^, Cu^2+^, EDTA	Ca^2+^, Ba^2+^, Na^+^, K^+^	[34]

CHIs are inducible (adaptive) enzymes, i.e., they are expressed only under certain conditions induced by a certain factor(s) and are regulated by a repressor/inducer system. While chitin is an inducer, glucose or another easily assimilable source of carbon may be a repressor [86]. Frändberg and Schnürer [27] maintain that the production of CHIs may be induced by chitin and chitooligosaccharides in the medium; however, no extracellular production of CHIs was observed in the absence of these compounds. Although some actinomycetes produce CHIs constitutively, an appropriate chitin substrate always increases production [77]. Inducing the production of CHIs through different chitin substances is a feature of a particular enzyme-producing strain. Numerous studies use colloidal chitin for the production of CHIs [44, 63]. However, some microorganisms can produce active CHIs in the presence of shrimp shells [10, 117]. The results of laboratory research aimed at evaluating the enzymatic activity of *Trichoderma* indicate that a medium containing purified chitin or mycelium as the sole source of carbon provides optimum growth conditions for inducing extracellular CHIs. Studies using different carbon substrates have confirmed the relationship between a metabolized source of carbon and the synthesis of chitinolytic enzymes. Chitinolytic activity was observed in bacteria cultured on media containing colloidal or glycol chitin, *N*-acetylglucosamine, chitooligosaccarides, or the cell walls of certain fungi. No or minimal activity was observed in the same bacteria cultured on media containing glucose or laminarin as the source of carbon [69, 88, 124]. Molecular studies have detected the presence of a two-component signal transduction system that regulates the synthesis of CHIs in *Pseudoalteromonas* *piscicida* O-7, *Streptomyces thermoviolaceus* OPC-520, and other bacteria. The systems consist of two proteins: a histidine kinase and a response regulator. In response to a signal from the environment (the presence of chitin or its derivatives), a bacterial kinase undergoes autophosphorylation at a histidine residue, then catalyzes the transfer of a phosphate group to an asparagine residue in the sequence of a response regulator. The phosphorylated response regulator is combined with a promoter sequence and activates the transcription of genes encoding CHI [87, 88, 111].

## Decomposition of Shrimp Waste, the Main Source of Chitin

The decomposition of chitin, an important part of the global carbon and nitrogen cycles [43], relies mainly on microbiological processes. Chitin can be used by microbial populations as the sole source of these two elements [67]. Considered common waste, shrimp waste (the main source of chitin) is processed for animal feed and is also used in agriculture as a cheap natural nitrogen fertilizer. However, it is not uncommon for shrimp waste to travel from a drainage basin to stagnant waters, where it is decomposed by different microorganisms. Swiontek Brzezinska et al. [106] determined the decomposition rate by evaluating oxygen consumption by the microorganisms present in shrimp waste. In this study, they used cephalic segments containing significant amounts of fat and protein and shells containing no fat or protein. The results of their study indicated that the decomposition rate was very high, with a relatively high oxygen consumption, which is typical of this type of environment. Due to the content of chitin and other components, the oxidation of shrimp shells was slower and less intense when compared to the oxidation of other parts.

In the water and bottom sediments of a highly eutrophic lake, shrimp waste was efficiently metabolized by planktonic and benthic microorganisms. Oxygen consumption in the presence of shrimp waste was higher in the bottom sediments than in the water, and this difference was associated with the higher accumulation of microorganisms in the sediment [107]. In addition, *Streptomyces rimosus* isolated from soil effectively metabolized not only shrimp waste but also chitosan, a chitin derivative. After culturing for 14 days, the decomposition rate of chitosan was 42.5 %, and the decomposition rate of shrimp shells was 38.2 %. Considering that these materials are difficult to decompose, the results can be regarded as satisfactory [105]. When Hoang et al. [43] studied the decomposition of shrimp shells by *Streptomyces* sp. TH-11, they noticed a substantial reduction in the weight of the shells as early as the 7th, 12th, and 16th days. Similar to other macromolecular compounds, chitin is not easily metabolized, even by chitinolytic microorganisms, because its oxidation requires time. According to Mudryk [72] and Grover and Chrzanowski [33], cellulose, similar in structure to chitin, is not widely used as a respiratory substrate, and only a small number of microorganisms can decompose this polymer.

Shrimp shell waste is tested primarily for the production of bioactive monosaccharides and oligosaccharides [18] such as *N*-acetylglucosamine, which is used for the production of cosmetics and nutritional supplements [12]. Shrimp shell is also a substrate for the production of CHIs. Actinomycetes and several species of bacteria use shrimp shells more effectively than colloidal chitin for the synthesis of CHIs. In addition, Rattanakit et al. [83] maintain that shrimp shell waste is a good substrate for culturing *Aspergillus* sp. S1–13, which, when grown on media containing shrimp waste, synthesizes the same or higher amounts of chitinolytic enzymes than the fungus grown on medium supplemented with colloidal chitin. Wang et al. [115], Chang et al. [10], and Wang and Chang [116] who investigated the chitinolytic activity of bacteria in the *Bacillus* and *Pseudomonas* genera used successfully shrimp and crab shell waste to produce CHIs.

## Microbiological CHIs in Biological Control

Storage fungi perform a very important role in the operation of ecosystems. As saprophytic organisms they degrade organic matter, leading to formation of humic substances. Nonetheless, many of these fungi are parasites and pathogens. The ones which attack crop plants are particularly dangerous. The most important plant pathogens include: *Fusarium*, *Penicillium*, *Alternaria*, *Botrytis*, *Ramularia*, *Monilinia*, *Cladosporium*, and *Aspergillus*. These molds attack many horticultural plants, including vegetables, fruit, and decorative flowers. In order to protect the crops people apply different types of crop protection chemicals (fungicides) produced in chemical synthesis. There are also many biological agents fighting fungal pathogens, containing various bio-active substances of microbiological origin, e.g., CHIs.

Abilities of microorganisms to produce antifungal CHIs are widely known (Table 2). The enzymes produced by the genus of storage fungi called *Trichoderma* are of significant biotechnological importance. The mechanism of their activities is well-known [5]. Application of preparations containing this mold in biological control of fungi development is possible as a result of production of such enzymes as CHIs or glucanases [2, 38, 58]. The exo-α-1.3-glucanase was able to bind to cell walls of various phytopathogenic fungi, such as *Aspergillus niger*, *Botrytis cinerea*, *Colletotrichum acutatum*, *Fusarium oxysporum*, *Penicillum aurantiogriseum*, or *Rhizoctonia solani* [2]. The CHI effectively inhibit the growth of *R. solani, Marchophominia phaseolina*, *Fusarium* sp. [4, 70, 75].

**Table 2:** Antifungal activity of some microbial CHIs

Source	Antagonistic against	References
*Trichoderma harzianum* Rifai TM	*Fusarium oxysporum f.* sp. *melonis*, *Sclerotium rolfsii*	[114]
*Trichoderma harzianum*	*Macrophomina phaseolina*, *Fusarium* sp. *R. solani*, *Aspergillus niger* (NCIM 563), *Aspergillus*, *Rhizopus*, *Mucor* sp.	[4, 70, 75]
*Trichoderma atroviride* PTCC5220	*Rhizoctonia solani*	[39]
*Trichothecium roseum*	*Alternaria alternata*, *Fusarium moniliforme, Magnaporthe grisea*	[22]
*Basidiobolus ranarum*	*Rhizoctonia solani*, *F. solani*.	[68]
*Bacillus* sp. BG-11	*Rhizopus arrhizus*, *Sclerotium rolfsii*, *R. solani, Phytophthora infestans*, *F. oxysporum*, *Phanerochaete chrysosporium*	[7]
*Bacillus cereus* YQQ 308	*Fusarium oxysporum*, *F. solani*, *P. ultimum*	[11]
*Bacillus pumilus* SG2	*Fusarium graminearum*, *R. solani*, *Magnaporthe grisea*, *Sclerotinia sclerotiorum*, *Trichoderma reesei*, *B. cinerea*, *Bipolaris* sp.	[28]
*Bacillus cereus* IO8	*Botrytis cinerea*	[36]
*Bacillus thuringiensis* subsp. colmeri 15A3	*Rhizoctonia solani*, *B. cinerea*, *P. chrysogenum*, *P. piricola*, *Penicillium glaucum*, *Sclerotinia fuckelian*	[61]
*Brevibacillus laterosporus*	*Fusarium equiseti*	[81]
*Aeromonas hydrophila* SBK1	*Aspergillus flavus*, *F. oxysporum*	[35]
*Enterobacter* sp. NRG4	*Fusarium moniliforme*, *Aspergillus niger*, *Mucor rouxi*, *Rhizopus nigricans*	[17]
*Alcaligenes xylosoxydans*	*Fusarium* sp. *Rhizoctonia bataticola*	[113]
*Stenotrohomonas maltophila*	*Fusarium solani*, *F. oxysporum*, *R. solani*, *A. alternata*	[46, 100]
*Rhizobium sp*	*Aspergillus flavus*, *Aspergillus niger*, *Curvularia lunata*, *F. oxysporum*, *Fusarium udum*	[97]
*Serratia marcescens* strain B2	*Botrytis cinerea*	[95]
*Vibrio pacini*	*Mucor racemosus*, *Trichoderma viride*, *Zygorhynchus heterognmus*, *Candida albicans*	[3]
*Streptomyces halstedii* AJ-7	*Alternaria alternata*, *B. cinerea*, *S. lycopersici*, *F. oxysporum*	[49]
*Streptomyces hygroscopicus*	*Colletotrichum gloeosporioides*, *Sclerotium rolfsii*	[80]
*Streptomyces tendae* TK-VL_333	*Aspergillus niger*, *F. oxysporum*	[51]
*Streptomyces* sp. DA11	*Aspergillus niger*, *Candida albicans*	[37]
*Streptomyces roseolus* DH	*Aspergillus* spp., *Rhizopus chinensis*, *Penicillium* spp., *Mucor* spp.	[47]
*Streptomyces sporovirgulis*	Alternaria alternata	[104]
*Streptomyces rimosus*	*Alternaria alternata*, *F. solani*	[105]

Moreover, CHIs of bacterial origin were also found out to show fungicidal properties. For instance, CHIs *Bacillus thuringiensis* spp. *colmeri* inhibit growth of many phytopathogens, including *R. solani*, *B. cinerea*, *Penicillium chrysogenum*, and *Physalospora piricola* [61]. Prasanna et al. [81] stated that CHIs from *Brevibacillus laterosporus* effectively inhibited development of *Fusarium equiseti*. These types of enzymes also showed insecticidal properties. Some yeast, e.g., *Pichia anomala* or *Pichia membranefaciens*, possess fungicidal properties as well. Their β-1.3-glucanases inhibited the growth of *B. cinerea* [48, 65]. Development of this mold was also effectively inhibited by the CHIs produced by the following yeast: *Candida saitoana, C. guilliermondii*, and *C. oleophila* [23, 89].

Actinomycetes also display strong fungicidal properties. It is related to the production of many types of various fungicidal compounds, including antibiotics and extracellular hydrolytic enzymes such as CHIs and β-1.3-glucanases [80]. *Streptomyces halstedii*, *S. griseus*, and *S. cavourensis* SY224 produce highly active antifungal CHIs, which implies the possibility of using them as agents for biological protection of crops [29, 49, 60].

With the use of genetic engineering techniques it is also possible to use in the biocontrol of phytopathogens the recombined enzymes obtained by introduction of proper genes encoding antifungal CHIs into various types of organisms. In the research performed by Bezirganoglu et al. [6] antifungal protein (AFP) and CHI fusion genes were introduced into oriental melons to control fungal diseases caused by *R. solani* and *F. oxysporum*. Their results demonstrated that the AFP–CHI fusion gene was effective in protecting the transgenic melon plants against fungal disease caused by *R. solani* and *F. oxysporum.* Matroodi et al. [66] have constructed a chimeric CHI by addition of chitin-binding domain from *S. marcescens* to the fungal CHI, *Trichoderma atroviride* Chit42, to study the role of chitin-binding domain in enzyme activity of Chit42. The evaluation of the antifungal activity of the constructed chimeric and native CHIs was performed to study the effect of ChBD in the antifungal activity of the chimeric CHI. Their study demonstrated that the fusion of ChBD improved the affinity to crystalline and colloidal chitin and also the enzyme activity of the chimeric CHI when compared with the native Chit42. The chimeric CHI showed higher antifungal activity toward phytopathogenic fungi.

Effectiveness of enzymatic agents in fighting fungal phytopathogens depends on many factors. Roberts and Selitrennikoff [85] discovered that CHIs of bacterial origin show considerably lower fungicidal activity than those of plant origin. The authors associated this phenomenon with the fact that the majority of bacterial CHIs are exochitinases whose activity is lower by one or two orders than that of endochitinases.

At the same time the fungicidal activity may be related to interaction of different types of enzymes. Research by Someya et al. [95] proved that synergic activity of endochitinase and chitobiase produced by *Serratia marcescens* inhibits the development of *B. cinerea* to a larger extent than either enzyme individually. Chang et al. [11] stated that *Bacillus cereus* QQ308 produced antifungal hydrolytic enzymes, comprising CHI, chitosanase, and protease, when grown in a medium containing shrimp and crab shell powder produced from marine waste. The growth of the plant-pathogenic fungi *F. oxysporum*, *F. solani*, and *Pythium ultimum* was considerably affected by the presence of the QQ308 culture supernatant. Certain genera of mold producing very strong endochitinases—*T. harzianum* and *Fusarium chlamydosporum* are of particular significance in protection of plants. Apart from chitnases, they also produce other hydrolytic enzymes, e.g., proteases, which perfectly interact with the chitin-decomposing enzymes [82].

Joo [49], testing fungicidal activity of purified and non-purified CHIs produced by *S. halstedii*, demonstrated that the enzymatic preparation inhibited the growth of several genera of fungal phytopathogens, to a greater or lesser extent. Purified CHIs inhibited development of *Alternaria alternata*, *Colletotrichum gloeosporioides*, *Fusarium oxysporium*, and *Stemphylum lycopersici* mycelia, and non-purified ones additionally caused the growth inhibition of *Phytophtora capsci*. Swiontek Brzezinska et al. [103, 104] found out in plate tests that the level of inhibition of the phytopathogenetic growth depended on the purity level of the enzymatic preparation. The CHIs which were not subject to purification turned out to be more powerful antagonists than the purified CHIs. It is probably connected with the presence of non-purified antibiotics which increase the fungicidal activity of the agents. Synergic interaction between β-1.3-glucanases and peptide antibiotics (peptabols) was revealed in the process of parasitism of *T. harzianum*. Peptabols and trichorzianines A and B are capable of inhibiting β-glucan synthetases (enzymes responsible for the synthesis of β-glucan in the cell wall) isolated from the plasmalemma of *B. cinerea*. The effects of peptabols may be strengthened by addition of β-1.3-glucanases produced by *T. harzianum*, which increases their fungicidal effectiveness [64, 120].

## Problems Associated with the Use of Microorganisms and Their Metabolites in Plant Protection Against Fungal Diseases

Biopreparations offer an alternative, environmentally friendly strategy for controlling phytopathogens. However, due to several challenges, their application is still limited. Although highly beneficial, the use of microorganisms and their metabolites also has some disadvantages. First, compared to chemical products, biological fungicides require more time to work. Moreover, due to their high selectivity, the costs associated with their use are relatively high [126]. The environmental conditions, which determine the growth of microorganisms, must also be considered. However, there are many ways in which different biofungicides can be combined or implemented using agricultural, physical, and chemical methods to produce a synergistic effect [57, 96].

Dahiya et al. [16] believe that chitinolytic enzymes can be used as supplements for chemical fungicides to increase their effectiveness against pathogenic molds and reduce the required concentrations of these harmful chemicals. In agriculture, the reluctance to use fungicides based on CHIs is associated with the fear that their impact will be reduced in the natural environment. However studies have shown that the combination of two chitinolytic bacteria *Paenibacillus* sp. 300 and *Streptomyces* sp. 385 is more effective against *F. oxysporum* causing cucumber wilt than individual strains or other combinations [94]. According to El-Tarabily et al. [24] the combination of *S. marcescens, Streptomyces viridodiasticus*, and *Micromonospora carbonacea* strains effectively inhibited the growth of *Sclerotinia minor* responsible for vegetable rot. Kishore et al. [54] described a synergistic effect of *Bacillus circulans* GRS 243 and *S. marcescens* GPS 5, the combination which inhibited the growth of the mold fungus *Phaeoisariopsis personata* when used as a prophylactic on the leaves. Forest protection relies on biofungicides containing *Trichoderma* species (mainly *T. viride* and *T. harzianum*), whose spores effectively inhibited the growth of fungi causing canker when mixed with beech sawdust and applied to the soil [50]. Agriculture relies on biofungicides containing various microorganisms (e.g., *Bacillus subtilis* GB03, *Pseudomonas aureofaciens* Tx-1, and *Streptomyces griseoviridis* K61), which are sold under different trade names [15]. The majority of biofungicides rely on colonizing the rhizosphere with microorganisms. By producing fungicidal enzymes and other metabolites, these microorganisms inhibit the growth of pathogens and protect plant roots.

## Conclusions

Chitinolytic microorganisms for a potential biotechnological application may be produced in various natural environments. Their application is not limited to degradation of the waste containing chitin. Numerous studies demonstrated the possibility of using them in production of chitinolytic enzymes with fungicidal activity against some fungal phytopathogens. In biological control of fungal phytopathogens, application of agents containing various metabolites of microorganisms, including CHIs, appears to be the more efficient, since they show stronger fungicidal activity than purified chitinolytic enzymes. The use of agents constituting a consortium of chitinolytic microorganisms seems to bring better results in fighting fungal phytopathogens. 
